# 68Ga-PSMA PET/CT in prostate cancer patients – patterns of disease, benign findings and pitfalls

**DOI:** 10.1186/s40644-018-0175-3

**Published:** 2018-11-01

**Authors:** Zohar Keidar, Ronit Gill, Elinor Goshen, Ora Israel, Tima Davidson, Maryna Morgulis, Natalia Pirmisashvili, Simona Ben-Haim

**Affiliations:** 1 Department of Nuclear Medicine, Rambam Health Care Campus, Haifa, Israel; 2 The Bruce Rappaport Faculty of Medicine, Technion – Israel Institute of Technology, Haifa, Israel; 3 Department of Nuclear Medicine, Wolfson Medical Center, Holon, Israel; 4 Sackler School of Medicine, Tel Aviv University, Tel Aviv, Israel; 5 Department of Nuclear Medicine, Chaim Sheba Medical Center, Ramat Gan, Israel; 6 Department of Medical Biophysics and Nuclear Medicine, Hadassah University Hospital, Ein Kerem, Jerusalem, Israel; 7 University College London and UCL Hospitals, NHS Trust, London, UK

**Keywords:** Prostate cancer, 68Ga-PSMA, PET/CT

## Abstract

**Background:**

68Ga-PSMA PET/CT has an important role in assessment of prostate cancer patients with biochemical recurrence and is evolving in staging high- and intermediate risk disease. The aim of present study was to describe the metastatic patterns and frequency of involved sites of prostate cancer and to assess the incidence of benign Ga68-PSMA avid PET/CT findings in a large patient population.

**Methods:**

68Ga-PSMA PET/CT studies performed in two tertiary medical centers over a period of 24 months were retrospectively reviewed. The incidence and location of pathological 68Ga-PSMA avid foci, suspicious to represent malignancy, as well as those of unexpected benign foci of increased 68Ga-PSMA activity were documented and analyzed.

**Results:**

There were 445 68Ga-PSMA studies in 438 men (mean age 72.4, range 51–92 years) with prostate cancer referred for biochemical failure (*n* = 270, 61%), staging high-risk disease (*n* = 112, 25%), response assessment (*n* = 30, 7%), follow-up (*n* = 22, 5%) and suspected bone metastases (n = 11, 2%). 68Ga-PSMA avid disease sites were observed in 319 studies (72%), in 181 studies (67%) for biochemical recurrence, 94 studies for staging (84%) (*p* < 0.05), in 22 studies for response assessment (73%), 10 follow up studies (45%) and in five patients with suspected bone metastases (45%). 68Ga-PSMA avid lesions were most commonly detected in the prostate (*n* = 193, 43%), loco-regional spread (*n* = 51, 11%), abdomino-pelvic nodes (*n* = 129, 29%) and distant metastases (*n* = 158, 36%), including bone metastases (*n* = 11, 25%), distant lymphadenopathy (*n* = 29, 7%) and other organs (*n* = 18, 4%). Distant 68Ga-PSMA-avid metastases were commonly seen in patients with biochemical recurrence (14/21 lesions), but were not seen in patient referred for staging (*p* < 0.013). There were 96 non-malignant 68Ga-PSMA avid foci in 81 studies, most common in reactive lymph nodes (*n* = 36, 38%), nonmalignant bone lesions (*n* = 21, 22%), thyroid nodules (*n* = 9, 9%), ganglions (n = 9, 9%) and lung findings (*n* = 8, 8%).

**Conclusion:**

The distribution of 68Ga-PSMA avid metastatic lesions is similar to data previously reported mainly from autopsy with comparable detection rates, indicating 68Ga-PSMA PET/CT is an accurate detection tool in patients with metastatic prostate cancer. If confirmed by further prospective studies 68Ga-PSMA PET/CT should be included in the guidelines to evaluate disease extent in these patients.

## Introduction

Prostate cancer is the most common solid malignancy in men and the 3rd leading cause of cancer related death with estimated 161,360 new cases and 6730 estimated deaths in the United States in 2017 [1, 2]. The diagnosis of prostate cancer is obtained by biopsy after clinical, biochemical or imaging suspicion arises [2]. Staging is determined by histology and using imaging modalities, mainly CT and MRI. Patients are then stratified into three risk level groups according to their stage, PSA level and Gleason Score (GS). Metastatic prostate cancer has a recognizable pattern of spread, involving mainly regional lymph nodes (predominantly pelvic and para-aortic) and the skeleton (predominantly the spine) [3]. Additional extranodal metastatic sites occur in the lungs and liver [3, 4]. Treatment strategies of prostate cancer are based on the patient’s risk group and include watchful waiting, hormonal therapy, radiotherapy, surgical intervention, chemotherapy or a combination of the above.

Prostate-specific membrane antigen (PSMA) is a type II transmembrane protein that acts as a glutamate carboxypeptidase enzyme [5, 6] and is a useful target for diagnostic and therapeutic applications in nuclear medicine because of its’ high expression in prostate cancer cells. The physiologic biodistribution of radiolabeled PSMA, at present mainly using 68Ga, includes the salivary and lacrimal glands, the small intestine, liver and spleen. It can also be taken up, to a lesser extent, in normal prostate tissue [5, 6]. 68Ga-PSMA is a valuable tool in the assessment and management of advanced prostate cancer patients. However, a wide range of malignancies other than prostate cancer have also been reported to express PSMA as part of tumor neovasculature [7–9] with 68Ga-PSMA avidity described in cases of breast cancer [10], renal cell carcinoma [11], glioblastoma multiforme [12], hepatocellular carcinoma [13], differentiated thyroid cancer [14], colorectal carcinoma [15], non-small cell lung cancer [16] and follicular lymphoma [17]. PSMA uptake has been also reported in a large variety of benign lesions such as retroperitoneal schwannoma [18], desmoid tumor [19], Paget’s disease of bone [20], sarcoidosis [21], sub-acute stroke [22] and bone fractures [23, 24]. Uptake in benign processes as well as in the celiac ganglia can mimic a lymph node metastasis [24, 25] and can therefore be pitfalls in clinical practice.

The aim of present study was to describe the metastatic patterns and frequency of involved sites of disease in prostate cancer and also to assess the incidence and outline the characteristics of benign Ga68-PSMA avid PET/CT findings in a large patient population.

## Materials and methods

### Study population

All 68Ga-PSMA PET/CT studies performed in two academic centers (RHCC and CSMC) over a 24-month period were retrospectively analyzed. In both centers the routine follow-up for patients with localized disease includes clinical follow-up every 3–6 months with PSA levels tested twice a year and digital rectal examination performed once a year. When PSA values increase, 68Ga-PSMA PET/CT is performed. In patients with metastatic disease, clinical follow-up is performed every 1–3 months including PSA levels and other blood tests. Bone scan and CT are performed every 3–6 months. 68Ga-PSMA PET/CT is performed when either radio ligand therapy using lutetium-177 PSMA or radiation therapy are considered. Patients’ charts were extracted from the institutional database and were reviewed. The ethics committees of both centers approved this retrospective data analysis and patient consent has been waived. The following clinical data were retrieved and recorded: age, indication for 68Ga-PSMA PET/CT imaging, GS and PSA level at the time of diagnosis and at the time of the PET/CT study. Previous therapy administered prior to the PET/CT study was also recorded.

### PET/CT acquisition and processing

PET and contrast enhanced CT (when not contraindicated) were acquired consecutively from head to the mid-thigh using a PET/CT system (Discovery 690, GE Healthcare, Milwaukee, US or Gemini XL, Philips Medical Systems, Cleveland, OH, US), approximately 60 min after the injection on average of 159 MBq (4.3 mCi) 68Ga-PSMA (range: 74 to 219.4 MBq, 2 to 5.9 mCi).

The following parameters were used for CT imaging: pitch 1.375:1, gantry rotation time 0.7 s, 120 kVp, automatically adjusted current in the range 100–650 mA, and a 2.5 mm slice thickness. A contrast enhanced CT scan was obtained 60 s after injection of 2 mL/kg of non-ionic contrast (Omnipaque 300; GE Healthcare). A PET scan followed in 3D acquisition mode for the same axial coverage. CT images were used for fusion with the PET data. PET images were reconstructed with CT attenuation correction using a 3D ordered subset expectation maximization (3D-OSEM) or a line of response row-action maximum-likelihood (LOR-RAMLA) algorithm.

### Interpretation and analysis of PET/CT images

All studies were reviewed retrospectively with knowledge of the patient’s clinical history and results of previous imaging studies. A team of two Nuclear Medicine physicians or a Nuclear Medicine Physician and a Radiologist interpreted the PET/CT studies in consensus. Any focal 68Ga-PSMA uptake higher than surrounding activity not associated with a known site of physiological uptake and with a corresponding morphological abnormality on CT was considered pathological and suspicious for malignancy. The fraction of 68Ga-PSMA PET/CT studies that were concluded as positive for malignancy was defined as “detectability rate”. Any site of incidental 68Ga-PSMA uptake considered to represent non-malignant findings was separately documented. The incidence and location of pathological 68Ga-PSMA avid foci, suspicious to represent malignancy, as well as those of unexpected benign foci of increased 68Ga-PSMA activity were documented and analyzed. Findings were characterized as malignant or as benign based on clinical correlation and other imaging modalities, when available.

#### Statistical analysis

Differences between average PSA levels in the study groups were assessed using the parametric Mann-Whitney test. Difference in detectability rates as well as in disease distribution between patient groups, categorized according to referral indications, were assessed using the Chi square and Fisher tests. *P* value smaller then 0.05 was considered statistically significant.

## Results

Four hundred and forty-five 68Ga-PSMA studies were performed in 438 men (mean age 72.4, range 51–92 years) with prostate cancer. Average GS was 7.5 (range 5–10). Average PSA level at diagnosis was 46.9 ng/mL (range 0–4000, median 11.0) and at the time of the PET/CT study 18.4 ng/mL (range 0.05–533, median 4.3). The indications for 68Ga-PSMA PET/CT included biochemical failure (*n* = 270, 61%), staging of high-risk disease (*n* = 112, 25%), assessment of response to anti-cancer therapy (*n* = 30, 7%), follow-up with no evidence of clinical, biochemical or imaging suspicious for recurrence on (*n* = 22, 5%) and suspected bone metastases on other imaging modalities performed as routine assessment (*n* = 11, 2%) (Table 1). Previous therapy, administered before PET/CT, is detailed in Table 1.

**Table 1:** Patient Characteristics, *n* = 445

Parameter	Value
Age	72.4 years (51–92 years)
PSA	
At diagnosis	46.9 ng/ml (0–4000 ng/ml, median 11.0)
At time of 68Ga-PSMA PET/CT	18.4 ng/ml (0.05–533 ng/ml, median 4.3)
Gleason Score	
< 6	50 (11.4%)
7	128 (29%)
> 8	146 (33%)
Average	7.5
Therapy prior to 68Ga-PSMA PET/CT	
Radical Prostatectomy	150 (34%)
Radiotherapy/Brachytherapy	171 (38%)
Hormonal	206 (46%)
Chemotherapy	23 (5%)
Other	27 (6%)
No prior treatment	122 (28%)
Indication for PET/CT	270(61%)
Biochemical failure	112 (25%)
Staging –high risk	30 (7%)
Assess response to treatment	22 (5%)
Follow up	11 (2%)
Suspected bone metastases	

**Fig. 1:**
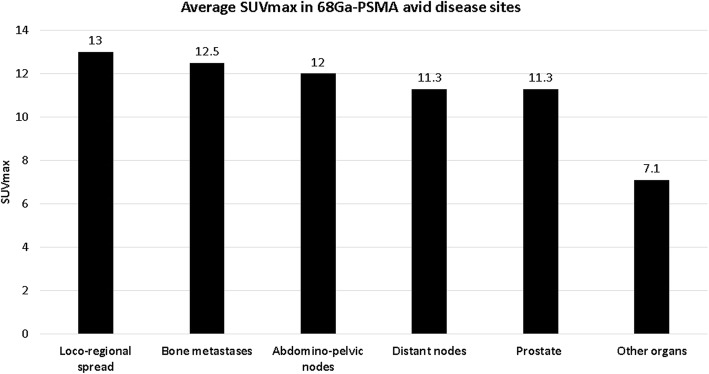
Average SUVmax in 68Ga-PSMA-avid disease sites

68Ga-PSMA avid sites of disease were detected in 319 studies (72%). Prostate gland involvement was detected in 193 studies (43%), loco-regional spread including seminal vesicles, bladder, rectum and adjacent fat tissue was seen in 51 studies (11%). Abdomino-pelvic nodal metastases were found in 129 studies (29%) and distant metastases including lymph nodes outside the abdomen and pelvis, bones and distant organs in 158 studies (36%) (Table 2). Radiotracer avidity (SUVmax) for different malignant sites is summarized in Table 2 and Fig. 1.

**Table 2:** Distribution of 68Ga-PSMA avid sites of prostate cancer involvement

	No studies (%)	SUVmax (range)
*N* = 319	72%	
Prostate	193 (43%)	11.3 (2–61)
Loco-regional spread	51 (11%)	13 (2–78)
Abdomino-pelvic nodal metastases	129 (29%)	12 (1.4–100)
Distant metastases	158 (36%)	
Bone metastases	111 (25%)	12.5 (1.9–91)
Distant nodes	29 (7%)	11.3 (1.5–58)
Other^a^	18 (4%)	7.1 (1.8–14.6)

^a^68Ga-PSMA avid metastases in lungs (*n* = 10, 48%), liver (*n* = 5, 21%), brain (*n* = 2, 10%), pleura (*n* = 2, 10%), spleen (*n* = 1) and peritoneum (*n* = 1)

68Ga-PSMA avid bone metastases were diagnosed in 111 studies (25%) including oligometastases (up to three lesions, *n* = 63, 57%) and multiple metastases (more than three lesions, *n* = 48, 43%).

Fifty-five 68Ga-PSMA avid lymph node metastases outside the abdomen and pelvis were identified in 29 studies (7%), including the mediastinum (*n* = 25, 45%), the cervical, supra- and infra-clavicular regions (*n* = 17, 31%), the axillae (*n* = 4, 7%) and additional thoracic sites (retro-pectoral, internal mammary, retro-crural; *n* = 9, 16%).

68Ga-PSMA avid metastases in other organs (21 lesions) were observed in 18 studies (4%). The distribution of these foci included the lungs (*n* = 10, 48%), liver (*n* = 5, 21%), brain (*n* = 2, 10%), pleura (n = 2, 10%), spleen (n = 1) and peritoneum (n = 1).

According to referral indications, none of the metastases in distant organs were found in patients evaluated at staging, whereas two thirds of these lesions occurred in patients who were investigated for biochemical failure (14/21, *p* < 0.013). 7 lesions were found in studies done for other indications (assess response to treatment, follow up and suspected bone metastases). There was no statistically significant difference between these groups in other sites of disease involvement including loco-regional spread (14% in staging vs. 10% in biochemical failure), abdomino-pelvic nodal metastases 32% vs. 30%), bone metastases (19% vs. 24%) and distant lymph nodes (4% vs. 7%).

The average PSA level in patients with disease limited to the prostate gland was 17.2 ng/mL compared to 28.9 ng/mL in patients with local or distant metastases (*p* = 0.2). The average PSA level in patients who were referred for staging of high risk disease was 29.9 ng/mL compared to 14.8 ng/mL in patients with biochemical failure (*p* = 0.05, borderline significant).

**Fig. 2:**
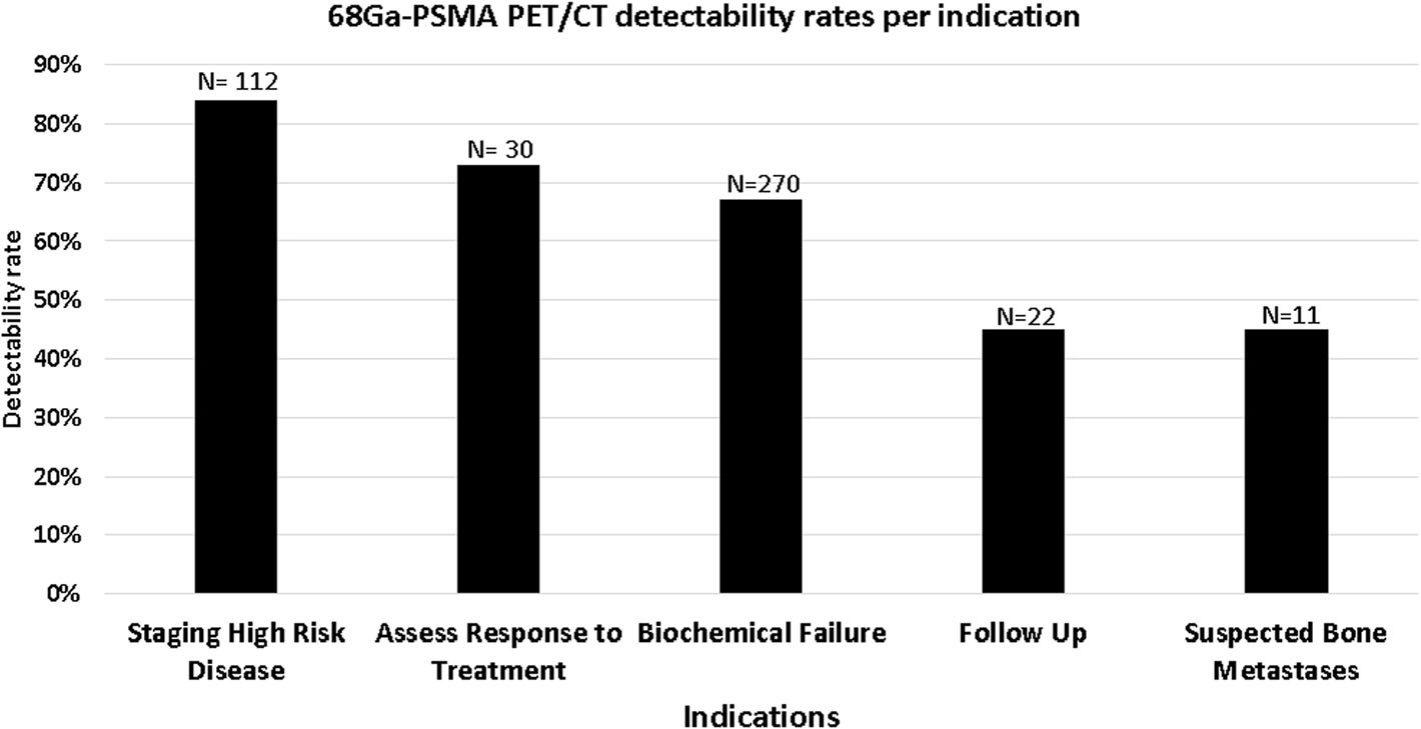
68Ga-PSMA PET/CT detectability rates per indication

Detectability rates of active disease using 68Ga-PSMA were calculated for different PSA levels and according to study indications. The detection rate was 31% for PSA 0–0.99 ng/mL, 63% for 1–1.99 ng/mL, 74% for 2–3.99, 77% for 3–9.99 and 90% in patients with PSA higher than 10 ng/mL. In 270 studies performed for the assessment of biochemical failure there were 181 (67%) positive 68Ga-PSMA-PET/CT studies. In patients referred for staging of high risk disease 94 out of 112 studies (84%) were positive. The difference in detectability rates between these two patient groups was statistically significant (*p* < 0.05). Detectability rates of malignancy in the additional patient groups were of 73% (*n* = 22) in cases assessed for monitoring response to treatment, 45% in patients were referred for follow up (*n* = 10), and 45% in patients with suspected bone metastases (*n* = 5) (Fig. 2). Due to small patient numbers in these subgroups the level of statistical significance could not be calculated.

**Fig. 3:**
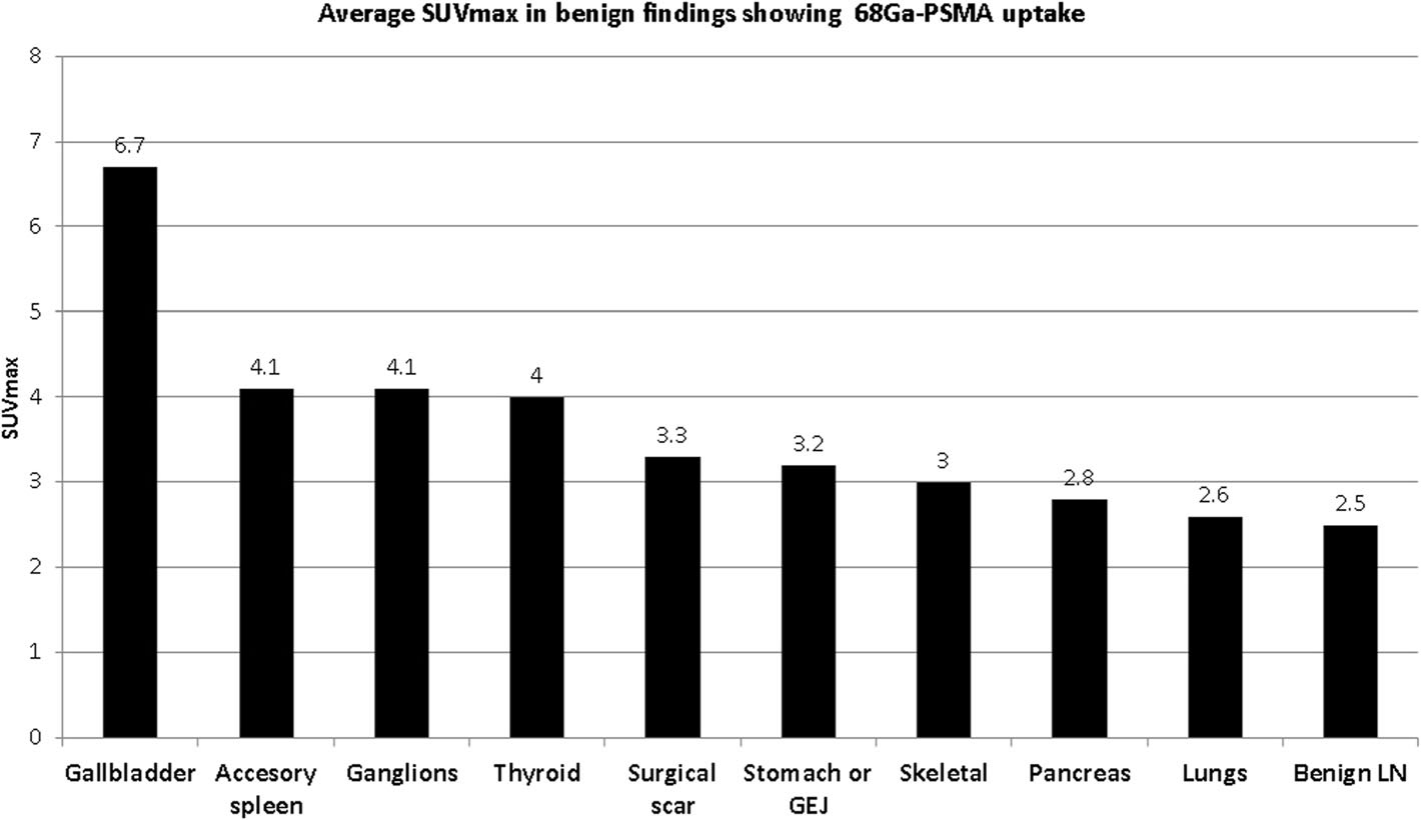
Average SUVmax in benign findings showing 68ga-PSMA uptake

Ninety-six 68Ga-PSMA avid foci were categorized as non-malignant in 81 studies (18%). They were localized to benign reactive lymph nodes (*n* = 36, 38%, in the axilla, inguinal region and pulmonary hila), the skeleton (*n* = 21, 22%, including 7 fractures, 13 degenerative changes and one patient with diffuse bone uptake related to known anemia), the thyroid gland (*n* = 9, 9%, including 6 with focal uptake in thyroid nodules and 3 with diffuse thyroid uptake), lungs (*n* = 8, 8%, including 6 opacities representing inflammatory infiltrates and 2 lung nodules), ganglions (n = 9, 9%, including 7 in celiac ganglion, one in stellate ganglion and one in a trigeminal ganglion), gallbladder (*n* = 5, 5%, without any specific CT findings), stomach or gastro-esophageal region (*n* = 4, 4%), pancreas (*n* = 2, 2%) and one case each in an accessory spleen and a surgical scar. Radiotracer avidity (SUVmax) of the non-malignant sites is summarized in Table 3 and Fig. 3.

**Table 3:** Distribution of 68Ga-PSMA-avid non-malignant findings

*N* = 96	No (%)	SUVmax (range)
Benign lymph nodes	36 (38%)	2.5 (0.7–17.7)
Skeletal Fracture Degenerative Anemia	21 (22%) 7 13 1	3 (1.4–5.5)
Thyroid Focal (nodules) Diffuse	9 (9%) 6 3	4 (2.3–9)
Lungs Inflammatory infiltrates Nodules	8 (8%) 6 2	2.6 (1.5–4.3)
Ganglions Celiac Other^a^	9 (9%) 7 2	4.1 (2.2–7.4)
Gallbladder	5 (5%)	6.7 (5.8–8)
Stomach or GEJ	4 (4%)	3.2 (3–3.76)
Pancreas	2 (2%)	2.8 (2.7–2.9)
Accessory spleen	1	4.1
Surgical scar	1	3.3
^a^One stellate ganglion, one trigeminal ganglion

## Discussion

68Ga-PSMA PET/CT has an important role in assessment of prostate cancer patients with biochemical recurrence [6, 26–31] and is evolving in staging high- and intermediate risk disease prior to surgery or radiotherapy [28, 32, 33].

In present study we have assessed the distribution of 68Ga-PSMA avid prostate cancer metastases in a large group of 445 patients from two tertiary medical centers referred mainly for biochemical recurrence (61% of patients) and staging of high grade disease (25%). The highest detectability rates of active disease were observed in these two groups of patients, 67% and 84% respectively. In a meta-analysis including 16 manuscripts and 1309 patients, the overall percentage of positive 68Ga-PSMA studies in biochemical recurrence was 76% (25), increasing from 42% in patients with PSA levels less than 0.2 to 95% when PSA was greater than 2 ng/mL (25). Detection rate of 67% in present study is comparable, taking into account the wide range of PSA values in the study population (0.05–533 ng/mL). In staging of high risk disease 93% of patients had intra-prostatic avidity whereas 68Ga-PSMA avid pelvic lymph node metastases were identified in 33.3% 4-20 mm in diameter. There were 66.7% false negative lymph nodes, measuring 1–10.8 mm in diameter (30). Another study in 51 patients with high risk disease reported sensitivity, specificity and accuracy of 53%, 86% and 76% in detecting lymph node metastases. Maximum length of tumor within the detected lymph node metastases was 5-30 mm, compared to 0.2-8 mm for undetected involved lymph nodes [34]. In another study in 42 patients with intermediate to high risk prostate cancer 68Ga-PSMA PET/CT identified all 41 involved lymph nodes with a short axis diameter > 10 mm as well as 8/10 involved lymph nodes with a short axis diameter of 5-10 mm. There were no involved lymph nodes with short axis diameter < 5 mm [35]. In other studies, neither extrapelvic lymph node metastases nor visceral involvement was found [33, 34]. Similarly, in present study there were no distant 68Ga-PSMA avid metastases in patients referred for staging of high risk disease.

Although the present study population differs, the distribution of metastatic lesions bears some resemblance to data previously published mainly from autopsy [3]. Bubendorf et al. have assessed the metastatic spread of prostate cancer in an autopsy study of 1589 patients [3], about half of them with previously known prostate cancer and the rest with an occult tumor. Metastatic prostate cancer was diagnosed in 35% of patients. In patients with lymph node metastases paraaortic nodes (in 80%) and pelvic lymph node metastases (55%) were most common, followed by mediastinal (40%) and inguinal nodes (18%) with only rare involvement of other nodal sites. Bone metastases, predominantly in the spine, were present in 90% of patients with metastatic disease, 46% had lung metastases, 25% liver and 21% pleural metastases. Rare sites of metastatic disease included the adrenals in 13%, peritoneum in 7%, meninges in 6%, kidney and ureter/urethra 3% each, pericardium and spleen 2% each and brain, thyroid, bowel, pancreas and mesentery in 1% each (*3*). In present study overall 72% of patients who were referred for various indications, all with known prostate cancer had 68Ga-PSMA avid disease. The most common sites of 68Ga-PSMA avid metastases included abdomino- pelvic nodal metastases in 29%, skeletal metastases in 25%, loco-regional spread in 11% and distant nodal metastases in 7% of cases. The most common distant nodal metastases were observed in the mediastinum, followed by cervical, supra- and infra-clavicular regions, with other nodal sites being less commonly involved. Other less common sites of 68Ga-PSMA avid metastases occurred in 4% of the studies, with almost half of them in the lungs, about 20% in the liver, and isolated cases of brain, pleura, spleen and peritoneal metastases. Interestingly, distant spread was most frequently seen in patients referred for the assessment of biochemical recurrence, but absent in patients referred for staging of high risk disease.

In present study 68Ga-PSMA avid bone metastases were diagnosed in 111 studies (25%). Focal 68Ga-PSMA uptake in the skeleton is usually considered to indicate the presence of bone metastases, unless it can be attributed to a corresponding skeletal lesion [18, 20, 23]. In current study, focal 68-Ga-PSMA uptake was also found in a variety of benign bone lesions. Caution and comparison with morphological findings on the CT component are therefore recommended prior to labeling, mainly solitary skeletal lesions, as metastatic. There is limited clinical evidence for the use of 68Ga-PSMA PET/CT in the evaluation of bone metastases in patients with prostate cancer. In a recent review of the published literature which included 31 case series and 6 case reports, 68Ga-PSMA PET/CT demonstrated higher diagnostic accuracy than bone scan in the initial staging and in biochemical recurrence, but not in patients with known metastatic prostate cancer [36].

Physiological distribution of 68Ga-PSMA as well as pitfalls and artifacts have been previously reported by several groups [5, 6, 10, 24, 25]. In the early days of using 68Ga-PSMA PET/CT, when the tracer was considered specific for prostate cancer, pitfalls in the interpretation of 68Ga-PSMA PET/CT studies were described, most of them as case reports [5, 17, 18, 20, 21], as well as case reports of 68Ga-PSMA avidity in other tumors (*9–15*).

To provide an accurate interpretation of 68Ga-PSMA PET/CT studies it is therefore important to be knowledgeable of the physiologic distribution of the tracer, the pattern of disease spread, as well as of avidity related to potential benign pitfalls. In present study 96 benign foci of 68Ga-PSMA avidity were found in a subgroup of 81 patients. Following numerous case reports this is, to the best of our knowledge, the first study to assess the prevalence and degree of 68Ga-PSMA avidity of non-malignant findings in a large group of patients with prostate cancer. Benign lymph nodes were the most common site of non-malignant 68Ga-PSMA activity, representing 38% of foci, followed by skeletal uptake, mainly in degenerative changes and fractures, in 22%. Up to 10% of findings included focal or diffuse uptake in the thyroid, in lung lesions or ganglions. In present study the prevalence of celiac ganglion uptake was considerably lower (7%) compared to Krohn et al. who identified focal celiac ganglion activity in 76 of 85 patients (89%) [25]. Increased gallbladder uptake was seen in 5% of patients, compared to 10% previously reported in a series of 40 patients [5].

Although on average the intensity of 68Ga-PSMA uptake in non-malignant findings was significantly lower as compared to that of metastatic lesions, there is a wide range of SUVmax levels. While some of the metastatic lesions presented with SUVmax of 2 or less some of the benign 68Ga-PSMA avid findings showed an SUVmax above 6. Therefore, it is important to be aware of the possible non-malignant etiologies of tracer uptake of even moderate or high intensity.

While in present series no other 68Ga-PSMA avid malignant tumors were identified this tracer has been shown to accumulate in other cancers, specifically in the endothelial cells of tumoral and peri-tumoral capillaries, possibly related to angiogenesis [8, 11–17].

The major limitations of present study are its retrospective nature and the lack of histopathological correlative data, the latter compensated by clinical and imaging follow up. However, since this study includes a large patient cohort assessed in two tertiary centers who were referred for different indications present findings could be used as a basis in planning of prospective studies.

## Conclusion

Present study shows 68Ga-PSMA avid sites of disease following the distribution pattern of prostate cancer involvement as previously described mainly in autopsy studies with comparable detection rates. 68Ga-PSMA PET/CT is therefore an accurate non-invasive tool. If current results will be confirmed by further prospective studies 68Ga-PSMA PET/CT should be included, in addition to MRI of the pelvis, in the recommendations and society guidelines to evaluate disease extent in patients with prostate cancer.

## Abbreviations

CSMC: Chaim Sheba Medical Center
CT: Computed tomography
Ga: Gallium
GE: General Electrics; OSEM: Ordered Subsets Expectation-Maximization
GS: Gleason score
MRI: Magnetic resonance imaging
PET: Positron emission tomography
PSA: Prostate specific antigen
PSMA: Prostate specific membrane antigen
RHCC: Rambam Health Care Campus
SUV: Standardized Uptake Value
